# Building a Beetle: How Larval Environment Leads to Adult Performance in a Horned Beetle

**DOI:** 10.1371/journal.pone.0134399

**Published:** 2015-08-05

**Authors:** Leeann T. Reaney, Robert J. Knell

**Affiliations:** School of Biological and Chemical Sciences, Queen Mary, University of London, Mile End Road, London, United Kingdom; Universidad Nacional Autonoma de Mexico, MEXICO

## Abstract

The link between the expression of the signals used by male animals in contests with the traits which determine success in those contests is poorly understood. This is particularly true in holometabolous insects such as horned beetles where signal expression is determined during metamorphosis and is fixed during adulthood, whereas performance is influenced by post-eclosion feeding. We used path analysis to investigate the relationships between larval and adult nutrition, horn and body size and fitness-related traits such as strength and testes mass in the horned beetle *Euoniticellus intermedius*. In males weight gain post-eclosion had a central role in determining both testes mass and strength. Weight gain was unaffected by adult nutrition but was strongly correlated with by horn length, itself determined by larval resource availability, indicating strong indirect effects of larval nutrition on the adult beetle’s ability to assimilate food and grow tissues. Female strength was predicted by a simple path diagram where strength was determined by eclosion weight, itself determined by larval nutrition: weight gain post-eclosion was not a predictor of strength in this sex. Based on earlier findings we discuss the insulin-like signalling pathway as a possible mechanism by which larval nutrition could affect adult weight gain and thence traits such as strength.

## Introduction

Research into the role of sexual selection as a driver of evolution has led to insights into many of the most fascinating aspects of mating system and reproductive biology [1–3]. In the case of sexually selected traits used in intrasexual selection, we know that males of many species either display sexually selected traits to each other during contests [4], as with the eyestalks of diopsid flies [5] and the dewlaps carried by *Anolis* lizards [6], or actively use them as weapons, examples of which include the antlers and horns carried by bovids [7] and beetles [8]. We also know that when two males carrying such structures compete it is often the one with the larger trait that wins, and that most of the time these sexually selected signal traits appear to be honest signals of the ability to win contests (often generalised to “resource holding potential” or RHP, [9,10] although not always [11].

Recent research which places an emphasis on functional morphology and on dynamic movement and related measures of performance, dubbed the “functional approach” to sexual selection [12,13] has started to shed light on how these signals might indicate RHP. In *Anolis* lizards, for example, males bite each other during fights and the size of the sexually selected signal, the dewlap, is correlated with bite force [14,15]. Male dung beetles engage their horns with each other and push against their opponents in tunnels, and horn length appears to be a better correlate of strength even than body size [16,17]. In these systems, then, it seems that at least one component of the RHP that is being signaled by the sexually selected trait is strength, which leads us to the question of how the link between signal and strength arises and is maintained.

Part of the answer lies in the observation that many sexually selected traits show “condition dependence”, meaning that the bearer’s overall health and well-being seems to have a disproportionate effect on sexually selected traits. This has been demonstrated experimentally by studies that have found that such traits respond more to aspects of the bearer’s environment and biology like diet or inbreeding than do other traits [18,19]. Over the last few years evidence has emerged which indicates that this condition dependence might be mediated by the insulin-like signaling (ILS) pathway. This pathway is one of the most important players in the control of growth and body size, and it has been shown that the sexually selected horns of the Japanese Rhinoceros Beetle, *Trypoxylus dichotomus* are considerably more sensitive to ILS than are other parts of the body such as wings or genitalia [19,20], with similar evidence now emerging from systems such as other beetle species, diopsid flies, deer and swordtail fish [19]. Given that the levels of insulin-like peptides (ILPs, the signalling molecules for this pathway in invertebrates) in an organism are closely linked to condition, tissues which are especially sensitive to the ILS pathway will show condition dependence as a consequence.

This ILS-based mechanism for condition-dependence could also explain the link between signal expression and strength in some animals, since the growth and maintenance of performance-related tissue such as muscle will also depend on resources from the environment, and it could be the case that the anatomical and metabolic traits linked to strength or other important aspects of performance respond to ILS in a similar way to the signal traits. In some animals, however, signal and strength develop at different life stages and are therefore not likely to be influenced by the same environment. In holometabolous insects, for example, the size of the signal trait is often determined during metamorphosis, either during the prepupal stage, as is the case with beetle horns [21], or the pupal stage, as in Diopsid flies [22] and is therefore influenced most by the larval environment. In many cases, however, these insects do not emerge from the pupa as a sexually mature adult, but undergo a “maturation feeding” period after eclosion during which time they grow significant amounts of soft tissue, meaning that the resources available for investment in musculature, and therefore strength, will be determined by the adult environment. To date, the biology and evolution of maturation feeding in insects has mostly been studied from the point of view of investment in reproductive traits, with a considerable amount now being known of the biology of egg maturation [23,24], alongside some studies of the effect of adult diet on male reproductive traits [25–27], but the link between larval environment, adult environment and performance traits is currently unknown.

Here, using the horned dung beetle *Euoniticellus intermedius* as a model organism we investigate the question of how resource availability during the larval and adult stages of an animal’s life determines the expression of a number of important traits, including signal size, body size, weight, strength and testes mass. Dung beetles are especially suitable animals for such experiments because the mother makes brood balls, each containing a single egg (in this species) so the resources available to the larva are strictly limited to those provided by the mother, meaning that it is simple to measure and to manipulate the larval resources available.

## Materials and Methods

*Euoniticellus intermedius* is a small (roughly 1cm long) dung beetle, originally from Southern Africa but now introduced into Australia and America. Females bury dung beneath pats to form brood balls, and lay a single egg in each one. The beetle larvae feed exclusively on the dung contained within a single brood ball, and following eclosion they engage in a period of maturation feeding that lasts approximately two weeks before they become sexually mature (Knell, unpublished observations). Male *E*. *intermedius* fight for access to females using a short horn on the clypeus [28], and horn size is closely correlated with both strength and endurance in these animals [16]. Body size and horn length of these animals is under both genetic and environmental control via resource availability to the developing larva but the latter seems to be considerably more important than the former, with heritability of horn length estimated at 0.12 in this species (Head, M. and Knell, R., unpublished). These beetles also have sexually dimorphic pronotums, with the top of the male pronotum extended forwards above the head (see figure A in S1 File).

For the experiment described here, freshly made brood balls were collected from a large laboratory culture that was originally started with animals collected in Queensland, Australia in 2007 and maintained at 28°C with a 12:12 h light: dark cycle using the methods described in [29]. In order to ensure that there was a good degree of variability in the amount of larval resources available, the brood balls were weighed using a Sartorius BP-221S balance and allocated at random to either a dung-removal or a no removal treatment. The former had 25% of their weight removed, thereby reducing the amount of food available to the developing larvae. The brood balls in the no removal treatment had 25% of their weight removed and then attached to the brood ball again.

Following manipulation, the brood balls were kept in damp sand in individual plastic pots and monitored until eclosion. The brood balls were weighed again once the animals emerged. The freshly emerged adults were weighed, sexed and allocated to one of two treatments: *ad libitum* access to dung, with fresh dung every five days, or restricted access with dung available for two days out of every five only, with damp cotton wool being provided for the remaining three days. Following a period of 16–18 days the beetles were weighed again and a measure of maximum strength was taken using the methods described in [16,17]. Briefly, a piece of cotton thread was attached to the rear of one elytron using cyanoacrylate adhesive. The beetle was allowed to enter an artificial tunnel made from two sheets of glass clamped together with two hardboard spacers, with sheets of fine sandpaper (300 grade) between the spacers and the glass to give a rough surface to grip on, making an artificial tunnel 3.5mm high and 6 wide. The tunnel was inclined at 60° from the horizontal, and the piece of cotton thread placed over a pulley and attached to a plastic pot. A gentle pull on the thread at this point causes the beetle to brace itself in the tunnel, and it will continue to do this until it is unable to resist the pulling force acting on it. To steadily increase the force, water was dripped into the plastic pot until the beetle was unable to resist the pull and came out of the tunnel. The force required to pull the beetle out of the tunnel was then measured by weighing the pot plus the water, and the weights were then converted to Newtons. Each beetle was tested three times with a rest period of between ten and fifteen minutes between each trial, during which time they were kept individually in plastic boxes with a piece of damp cotton wool. For the analysis here the highest measurement of pulling force for each beetle was used [30].

The beetles were then killed using ethyl acetate and photographed using a digital camera (Nikon Coolpix 950) mounted on a dissecting microscope, and the images taken used to obtain measurements for elytron length, pronotum length and horn length using ImageJ image analysis software (v 1.45, available from http://imagej.nij.gov/ij). The testes were dissected out of the male beetles, weighed, and placed back in the body of the animal. The overall fat content of each beetle determined by using a Soxhlet apparatus [31]. Fat content provides a measure of the overall energy stored by the animal, which is closely related to Rowe and Houle’s [32] original definition of “condition” as the total pool of resources available for allocation to fitness enhancing traits. Fat content is often therefore used as a measure of condition [33–35] and some authors have argued that fat content should be related to mating success [35,36] (NB Rowe and Houle’s definition does not explicitly mention “energy” although it is sometimes stated that it does, e.g. [37]). A total of 98 male and 93 female beetles were measured in this way.

### Data analysis

This experiment provided a series of measures of resource availability (brood ball weight, brood ball manipulation, adult food availability) throughout the animals’ lifetimes, and also a series of measures of important aspects of the animals’ size, performance and investment in somatic and reproductive tissue, including elytron length (a measure of overall body size), the length of the sexually dimorphic pronotum, horn length (males only), weight at eclosion, weight gain during maturation, testes mass (males only), strength and total fat content. Because some of the variables that were measured or manipulated early in the animals’ lives are likely to have indirect effects on traits expressed later on (for example, if body size is affected by brood ball weight, and testes mass is related to body size then brood ball weight will indirectly influence testes mass via body size), conventional linear modelling will not be able to describe the relationships between these variables fully. Following initial exploratory analysis we therefore analysed the relationships within the dataset by using path analysis [38], which has a number of advantages for modelling complex datasets of this nature [39]. For the purposes of this study, the chief benefits are that it allows indirect as well as direct effects to be estimated, and that it allows the easy construction of graphical diagrams showing the interrelations between variables.

Exploratory data analysis found that horn length was very closely correlated with pronotum length in males (Pearson’s r = 0.91). This means that including both of these explanatory variables in a model will cause problems due to multicollinearity and as a consequence path models were fitted with only horn length included.

All path models were fitted using the Lavaan package (v 0.5–17, [40,41] running in R v3.01. Following an initial model fit, a minimal model was produced by the removal of non-significant terms and the addition of terms that were not previously included when modification indices [38] suggested that the inclusion of these terms would lead to a significant increase in the model’s explanatory power. The data from males and females were analysed separately because females do not have horns or testes, but there are also some interesting contrasts between males and females and these were presented using the results from normal linear models.

Two measures of goodness-of-fit are presented in the results, both of which are in truth measures of “badness of fit” so values close to zero are indicative of a well fitted model. One is the minimum function test statistic, which is calculated as n-1 multiplied by the minimum value of the maximum likelihood estimator from the fit function, and the other is the Root Mean Square Error of Approximation (RMSEA). The former is a standard measure used in path analysis and which is usually expressed as the result of a chi-square test comparing the value to zero, so a high p-value is desirable. This measure is somewhat dependent on the sample size, however, so we also present the RMSEA which takes sample size into account. RMSEA values <0.05 are usually taken as indicating good fit, whereas RMSEA values >1 are suggestive of a poor fit. [38].

Some of the assumptions behind path analysis mean that care must be taken with path models. Firstly, and most importantly, the path model that is fitted is an explicit hypothesis of how causality works in the system being analysed: the effects, both direct and indirect, of one variable on another are assumed to be directional. This can clearly present problems in many situations, but when the variables are temporally ordered, as is the case here, this assumption is likely to be reasonable. Secondly, the relationships between variables are assumed to be linear and additive—in other words, if there are interaction effects they cannot be easily incorporated into path models. Here, we performed preliminary analysis by fitting linear models to our data to check for non-linear relationships and important interaction terms. No significant interaction terms were found and although there were some relationships that were best described by a quadratic relationship in a simple linear model (e.g. the relationship between weight gain and strength) these were not found to be important in the path models. Finally, path models do not work especially well with variables that only take a small number of discrete values, relevant examples in this case being sex (male or female), brood ball size (manipulated or unmanipulated) and adult diet (restricted or unrestricted). Sex was removed from the fitted models because we fitted separate models for the two sexes. Initial analysis found that while brood ball manipulation was important, almost all of the effect of manipulation was a consequence of the change in brood ball weight: whether the brood ball was manipulated or not only had an effect beyond this in the linear model fitted to explain elytron length in the preliminary analysis, and the effect here was small. As a consequence of this only the weight of the brood ball was included in the path model as an indicator of juvenile resource availability. Adult diet was included in the path models as a numerically coded variable, meaning that the path analysis treats it as a continuous variable. This is not especially satisfactory and is likely to lead to inflated parameter estimates for this particular variable [40], but given that the remaining variables are continuous and that preliminary analysis with simple linear models indicated that effect of this variable was small (a conclusion borne out by the path analysis results) we argue that this is the most straightforward way of dealing with this particular problem.

## Results

### Comparing the strength of male and female beetles

Although female beetles were slightly smaller than males with elytra lengths that were, on average 0.01mm shorter (t-test comparing elytra lengths, t = 3.48, 185df, p = 0.0006), female beetles were significantly stronger than males (t = 2.583, df = 166, p = 0.0107, test carried out using log-transformed values for strength to stabilize variances). On average, male beetles being able to resist a pulling force of 39.96N whereas females could resist one of 45.86N. This pattern remained when body size was controlled for by including elytra length in a model (Sex F1,165 = 20.5 p<0.0001, Elytra length F1,165 = 39.5, p<0.0001, see figure B in S1 File).

### Path analysis for strength

**Fig 1:**
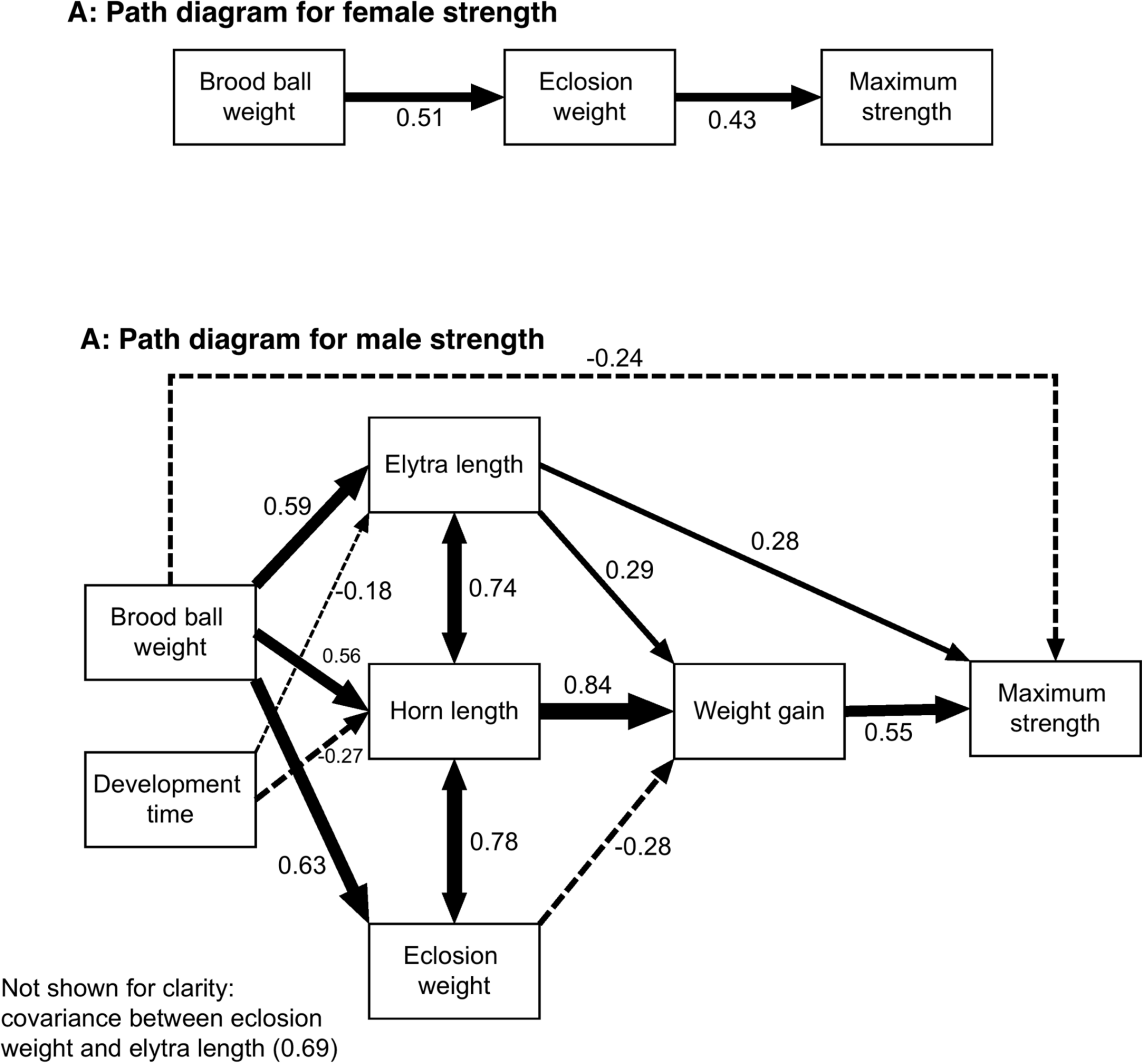
Path diagrams for the final models explaining the relationship between female (A) and male (B) maximum strength and larval nutrition, adult morphology and post-eclosion weight gain. Solid lines indicate positive relationships, dashed lines negative, double headed arrows indicate correlation (i.e. no assumptions about causality) and line width is proportional to the strength of the relationship. Numbers next to arrows indicate regression or correlation coefficients calculated from standardized predictor variables. Haemolymph protein content, overall fat content and adult diet treatment were not retained in either model and so are not shown.

Fig 1 shows the path diagrams for the final models following model refinement by the removal of variables which had little or no effect on strength and by adding in variables as indicated by modification indices. Full details of the analysis can be found in the online supplementary material, as can diagrams of the initial path models that were fitted (figures C and E in S1 File). The fit measures for the final path model for males indicate a well-fitted model [38], with a p-value for the minimum function test statistic of 0.553, which is far from 0.05, and a root mean square error of approximation (RMSEA) which is either zero or very close to it (estimate = 0, 95% CIs 0–0.118). The fit measures for the model for females are not quite as good (minimum function test statistic p = 0.190, RMSEA estimate = 0.08, 95% CIs 0–0.306) but are within satisfactory limits.

The final path model for female performance (Fig 1A) is straightforward: maximum strength is positively correlated with eclosion weight, which is itself positively correlated with brood ball weight. None of the other potential explanatory variables remain in the model. The path model for male performance (Fig 1B), however, shows a more complex pattern. There are direct positive effects of both elytra length (a measure of body size) and weight gain after eclosion on maximum strength, with weight gain having the largest effect, and a small negative direct effect of brood ball weight. There are also a number of indirect effects mediated via horn length and weight gain after eclosion: horn length, which is itself positively related to brood ball weight and negatively related to development time, is by far the strongest predictor of weight gain after eclosion. Elytra length, a measure of body size, also has a smaller indirect positive effect on maximum strength via weight gain following eclosion, and like horn length is positively influenced by brood ball weight and negatively by development time. Weight on eclosion has a small negative effect on weight gain. None of haemolymph protein content, overall fat content and adult diet treatment made a significant contribution to maximum strength for either male or female beetles and so none of these were retained in the final path models.

This complex pattern for male performance can be summarised as follows: horn size, body size (as indicated by elytra length) and weight on eclosion are strongly positively correlated with the amount of resources available to the developing larva (brood ball weight). Large beetles and beetles with longer horns develop slightly faster than small animals or those with short horns. Following eclosion, large beetles gain slightly more weight during maturation feeding but horn length is by far the most important predictor of weight gain. Beetles that were relatively heavy when they eclosed gain slightly less weight. Relatively large beetles are somewhat stronger, but the best predictor of strength is the amount of weight gained after eclosion.

Maximum strength is also negatively correlated with brood ball weight to a smaller degree, indicating that beetles which emerged from relatively heavy brood balls for their size and horn length tend to be slightly less strong.

### Fat content and testes mass

**Fig 2:**
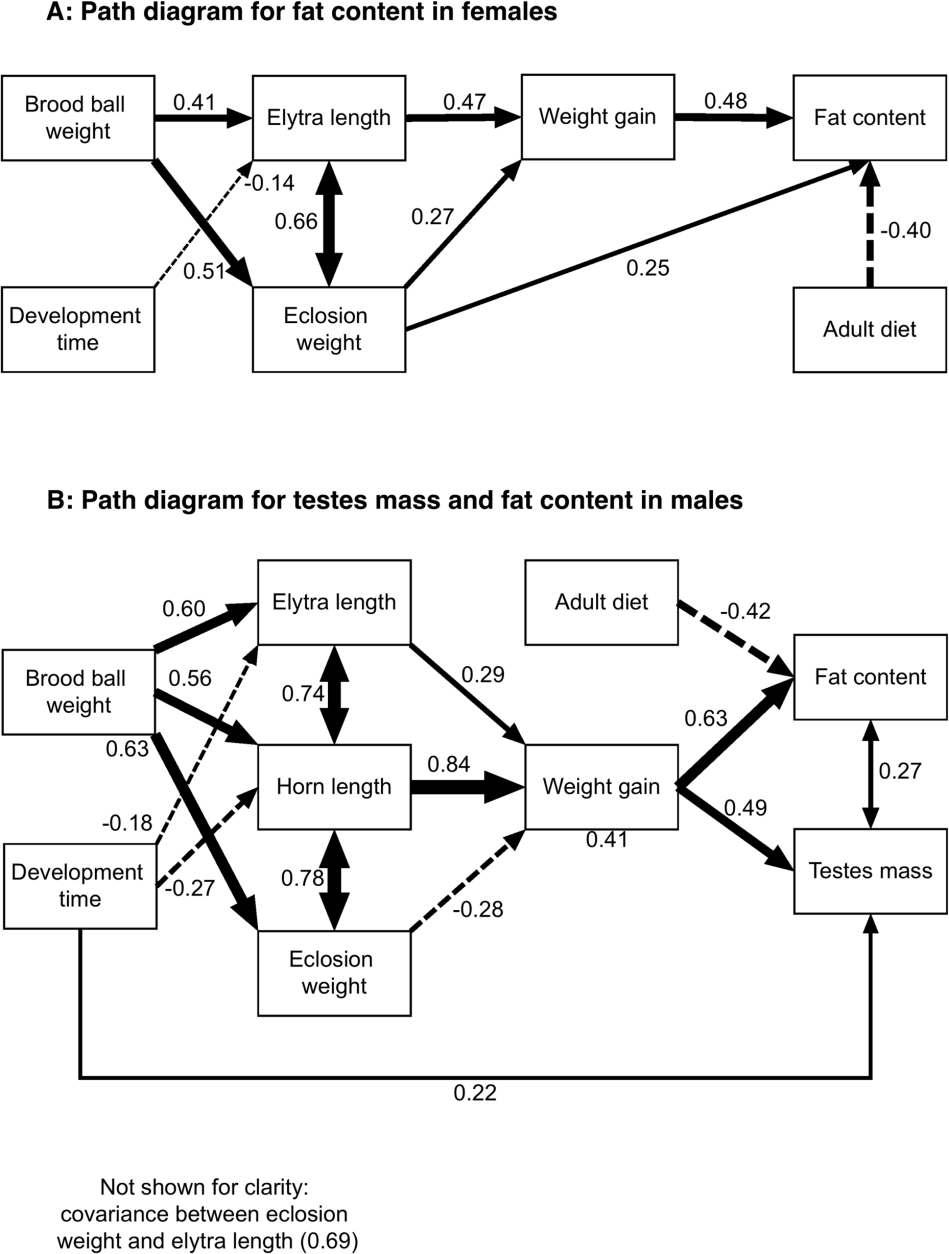
Path diagrams for the final models explaining the relationship between female (A) and male (B) adult fat content, testes mass (males only) and larval nutrition, adult morphology and post-eclosion weight gain. Solid lines indicate positive relationships, dashed lines negative, double headed arrows indicate correlation (i.e. no assumptions about causality) and line width is proportional to the strength of the relationship. Numbers next to arrows indicate regression or correlation coefficients calculated from standardized predictor variables.

The initial path models that were fitted to explain female body fat content and male body fat content and testes mass (see figures G and I in S1 File) were similar to the initial models used to explain maximum strength shown in Fig 1, with the differences being firstly that maximum strength for males was replaced with testes mass in the initial model, and for females this variable was removed. Secondly, the relationships between testes mass, fat content and haemolymph protein concentration were modeled as correlations rather than as causal relationships. Fig 2 shows the path diagrams for the fitted models following model refinement. In both cases the various fit measures indicate well-fitted models (females: minimum function test statistic p = 0.420, RMSEA 95% CIs 0–0.109, males: minimum function test statistic p = 0.823, RMSEA 95% CIs 0–0.135).

The final path model for female fat content (Fig 2A) shows the expected links between larval resource availability (brood ball weight) and both size (elytra length) and weight of female beetles. As with the path model for male strength, there is a small negative effect of development time on elytra length, indicating that larger beetles develop slightly faster than smaller ones. Both elytra length and weight at eclosion have indirect effects on fat content, mediated by weight gain during maturation feeding, and weight at eclosion also has a direct positive effect on fat content. The other important influence on fat content is adult diet: those animals receiving a restricted diet had a substantially reduced fat content.

When considering both testes mass and fat content in males (Fig 2B), weight gain after eclosion again occupies a central role in determining the values of both of these variables. The path model leading to weight gain is similar to that seen previously for maximum strength, with horn length, itself determined by larval resource availability and somewhat negatively related to development time, being by far the most important predictor of weight gain. As is the case with females, adult diet is related to fat content and animals given the restricted diet had significantly less fat content than those given an *ad-libitum* diet. Testes mass is positively correlated with fat content so there is an indirect effect of adult diet on testes mass but this is small because the correlation is weak. Finally, development time is weakly positively correlated with testes mass: large beetles and those with longer horns may develop faster, but once these factors are controlled for those animals that take longer to develop have larger testes.

## Discussion

The chief motivation behind this work was to answer the question of whether the horn of *E*. *intermedius*, a signal which is expressed in response to larval nutrition, can reliably give information about adult resource holding potential (RHP), which is determined at least partly by adult nutrition. Using path analysis we can see a more complete picture of the effects of larval and adult nutrition on these animals than could be achieved with conventional linear models, and we find striking indirect effects of larval nutrition on adult weight gain, fat content and performance. In males, larval food availability is important in determining horn length, which is itself the best predictor of determining weight gain during maturation feeding. Weight gain is then the single best predictor of strength, assumed to be closely linked to RHP and important in contests between males [16,17]. We also find a strong link between weight gain and testes mass, which will contribute to success in sperm competition [1]. Larval nutrition therefore affects both pre- and post-copulatory male fitness via post-eclosion weight gain, with horn length serving as a sensitive signal of the male beetle’s ability to assimilate nutrients and grow tissues associated with strength and sperm production as an adult.

Horn size is not, of course, the direct cause of adult weight gain, strength or testes mass. Rather, it seems likely that the same larval conditions that are largely responsible for determining horn size also control the adult beetle’s ability to assimilate resources and convert them into muscle, reproductive tissue and also fat. Considering musculature as an example, adult muscles in those insects that have been studied mostly develop *de novo* during metamorphosis from myoblasts which are specified during embryogenesis or, in the case of flight muscles, develop on scaffolds provided by larval muscles [42]. While the regulation of development of these muscles by substances such as the transcription factor Mef2 [43] and the GTPase Rab11 [44] is well described the mechanisms controlling the final size and performance of muscles are not currently well understood. One potential physiological link between larval nutrition and adult tissue growth comes from the recent work referred to in the introduction linking horn length to the ILS pathway [20]. The ILS pathway is of central importance in controlling growth in animals [19] and differential sensitivity to insulin/ILP levels is likely to be important in determining growth rates of a variety of tissues. If animals that receive poor nutrition during the larval period have low levels of circulating insulin/ILPs post-eclosion, and if both horns and the tissues that develop during maturation feeding are sensitive to insulin/IGF levels in a similar way then this would explain the link between these traits. Alternatively, the link between larval nutrition and adult development could be a consequence of ILS receptor numbers or sensitivity–if the expression of ILS receptor molecules in adult tissues is affected by the larval environment, for example, we could see the patterns observed here. In the case of muscles, therefore, it is possible that the myoblasts in the larvae respond to resource levels, potentially via the ILS pathway and that this response then affects growth of muscle tissue in the adults, but it is also possible that low levels of circulating insulin or ILPs in adults that are a consequence of poor larval nutrition are controlling muscle development in these insects.

The link between signal expression and performance can be broken by dietary manipulation in *Anolis carolinensis* lizards [45] and in adult horned “major” males of the dung beetle *Onthophagus taurus* [17]. In this experiment, however, and in contrast with larval diet, adult diet only had direct effects on fat content in both males and females, had no discernible impact on the strength of males or females, and only a weak indirect effect on testes mass via its effect on fat content. It is useful to compare the poor adult diet treatments in the present study with the one described by Knell and Simmons [17]. The experiment described in the current paper was designed so that the degree of food deprivation that the beetles on the restricted diet were exposed to was not severe, and they had access to food for two days out of every five. In the other experiment described by Knell and Simmons, however, three treatments were used; ad-lib dung, poor-quality dung which had had most of the liquid content removed, and no food at all for five days, and it is notable that in the Knell and Simmons study only the no-food treatment affected performance: although the poor-quality dung treatment had an effect on beetle weight it had no measurable effect on performance, a similar result to the findings described here. It seems that when food is scarce these beetles preferentially invest in traits which are critical to their fitness, such as strength and testes mass, at the expense of their fat storage, and only cease investing in strength under starvation conditions.

The contrast between the path models for strength for female and male beetles is striking. While strength in male beetles is determined by a complex web of direct and indirect effects, the final path model for female strength is especially simple: strength is determined by eclosion weight, which is itself determined by brood ball weight. Larval resource availability therefore influences female strength, but no post-eclosion variables seem to be important, suggesting that perhaps the growth of muscle tissue during maturation feeding in these animals is sex-specific. Interestingly, female beetles were also found to be stronger than males in this experiment. This may seem counter-intuitive at first sight, but it should be remembered that the female beetles of this species carry out all of the work during brood ball construction: we have never observed males assisting in this process, unlike some other species of dung beetle [46]. From a developmental point of view this disparity is interesting because it implies that there must be sex-based differences in the development of these tissues: it is possible that in adult females the developing muscles do not respond to signals in the same way that they do in males, for example. A further possibility is that there could be sex differences in the muscle metabolism in these animals due to the differing metabolic requirements of males and females, as has been described in hawkmoths [47] and these could lead to simpler effects leading to the final performance of the individual.

One notable aspect of the path analyses carried out here is that there is no suggestion of trade-offs between body parts. Several previous studies have found evidence that horned beetles trade off horns against other body parts including eyes, antennae, wings [48] and genitals [49]. Most relevant to the present results is a study of *Onthophagus nigriventris* which found that cauterising the tissue that would develop into horns in males of this species led to the growth of larger testes [50]. No such trade off can be seen here, with horn length being indirectly positively correlated with testes mass as a consequence of its relationship with weight gain. There are a number of possible explanations for this disparity. Most obviously, Simmons and Emlen [50] removed horns completely from their experimental animals, whereas we are only looking at correlations, making it hard to compare results directly. Secondly, the horns of *O*. *nigriventris* are considerably larger in relation to body size than those of *E*. *intermedius*, so the extra resources required for their construction might trade-off against those available for testes development in a way that doesn’t happen in *E*. *intermedius*. Finally, *O*. *nigriventris* testes might undergo less development post-eclosion than do those of *E*. *intermedius*. If most of the mass of the testes of *O*. *nigriventris* is laid down during metamorphosis then a trade-off against horn size would be more likely.

In the final path models, development time was not determined by brood ball weight, but it was negatively related to elytra length and, in males, to horn length meaning that for a given amount of food those animals that developed faster were larger and, in the case of males, had longer horns. This is again somewhat at odds with previously reported data from horned beetles–in particular, Hunt and Simmons [51] reported that development time was positively correlated with the amount of dung available to developing male *Onthophagus taurus*, but not to females. Given that male *O*. *taurus* are dimorphic, with those animals reared in large brood balls usually developing into large, horned “major” males whereas those that grow in small brood balls tending to develop into hornless “minor” males, Hunt and Simmons inferred that the requirement to grow larger horns explained the longer development times seen in this species when there was a lot of dung available–the opposite of what’s observed here. *E*. *intermedius* males are not dimorphic so the differences in horn length between large and small males are less extreme than in *O*. *taurus*, and in *E*. *intermedius* it might simply be the case that those animals which are generally more efficient at converting dung into beetle are able both to emerge more quickly and to grow larger than others.

Total fat content was affected by both larval and adult nutrition, but while it was associated with testes mass it was unrelated to strength in either males or females. This indicates that in *E*. *intermedius* at least total fat content is likely only to be an indicator of certain fitness-related aspects of an animal’s biology. The use of “condition indices” and also of lipid content as a general indicator of fitness or quality has recently been criticized on the grounds that (among other things) fat content is not necessarily always an indicator of fitness [34], and our date offer further support for this argument.

Post-eclosion feeding, often called “maturation feeding”, has historically been studied largely in terms of its relationship with female reproductive output. This study demonstrates that maturation feeding is also centrally important in determining male fitness, and that in *E*. *intermedius* at least the effect of maturation feeding can itself be a consequence of larval resource availability. Future studies of resource-mediated trade offs and of important traits such as performance and testes mass in insects will need to consider maturation feeding as a potentially important factor, and should ideally examine the effects of nutrition throughout an animal’s life cycle rather than only during a single life stage.

## Supporting Information

S1 File Analysis details and code. Full details and code for the path analysis and other analyses described in the paper. Includes ten figures including exploratory analysis (figures A&B) and diagrams for all initial and final path models (figs C-J).
(PDF)
