# Supplementary material for: Building a Beetle: How Larval Environment Leads to Adult Performance in a Horned Beetle
Source: PLoS One. 2015 Aug 5;10(8):e0134399. doi: 10.1371/journal.pone.0134399 (PMC4526545; doi:10.1371/journal.pone.0134399)
Supplement: S1 File — Full details and code for the path analysis and other analyses described in the paper. Includes ten figures including exploratory analysis (figures A&B) and diagrams for all initial and final path models (figs C-J). (PDF) [file pone.0134399.s001.pdf]

Supplementary material for “Building a beetle: how larval environment leads to adult fitness in a horned beetle”

Rob Knell & Leeann Reaney
School of Biological and Chemical Sciences
Queen Mary University of London

24 July, 2015

Contents

1 Introduction **2**

2 Sexual dimorphism in pronotum length **3**

3 Comparisons of male and female beetles **4**

4 Path analysis for male performance **7**

4.1 Data transformations used: 7
4.2 Initial model 7
4.3 Final model 10
4.4 Notes 12

5 Path analysis for female performance **12**

5.1 Data transformations used: 12
5.2 Initial model 13
5.3 Final model 15

6 Path analysis for male testes mass and fat content **17**

6.1 Initial model 17
6.2 Final model 20

7 Path analysis for female fat content **24**

7.1 Initial model 24
7.2 Final model 26
7.3 Notes 28

References **28**

1 Introduction

This document contains supplementary information and full details of the analysis, including all the R (R Core Team 2014) code necessary, for the paper named above.To run the R code below you will need the lavaan (Rosseel 2012), scales and ggplot2 (Wickham 2009) packages installed, and the data files Beetle_data_exploratory.txt, maledata.txt and femaledata.txt which can be obtained from the Dryad archive. This document was prepared in R markdown and rendered to pdf using the rmarkdown v2 package.

2 Sexual dimorphism in pronotum length

```
Beetle <- read.table("Beetle_data_exploratory.txt", header = TRUE)
library(ggplot2)
library(scales)
attach(Beetle)
p1 <- ggplot(data = Beetle, aes(x = Elytra, y = Pronotum, shape = Sex,
    colour = Sex))
p1 <- p1 + geom_point() + scale_shape_manual(values = c(16, 17)) +
    scale_color_manual(values = c("darkred", "steelblue")) +
    theme_bw()
p1 <- p1 + xlab("Elytra length (mm)") + ylab("Pronotum length (mm)")
p1
```

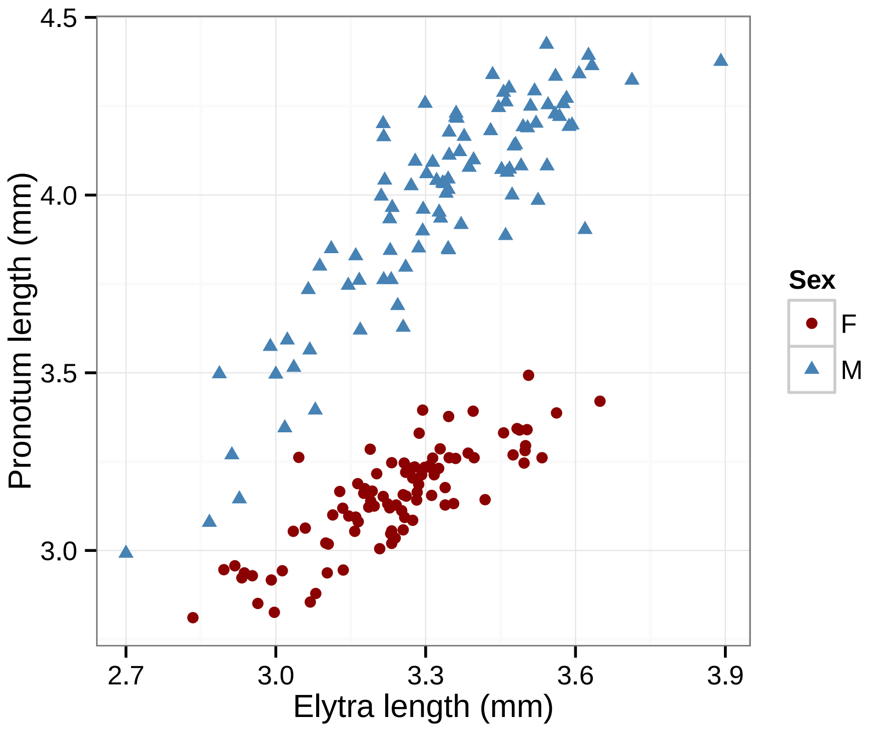

Figure 1:

Figure A: Pronotum length plotted against elytra length for male and female beetles.

3 Comparisons of male and female beetles

```
p1 <- ggplot(data = Beetle, aes(x = Elytra, y = MaxPerform, shape = Sex,
    colour = Sex)) + geom_point()
p1 <- p1 + scale_shape_manual(values = c(16, 17))
p1 <- p1 + scale_y_continuous(breaks = c(20, 30, 40, 50, 60,
    70, 90), trans = log_trans())
p1 <- p1 + scale_color_manual(values = c("darkred", "steelblue")) +
    theme_bw()
p1 <- p1 + xlab("Elytra length (mm)") + ylab("Maximum strength (N)")
p1
```

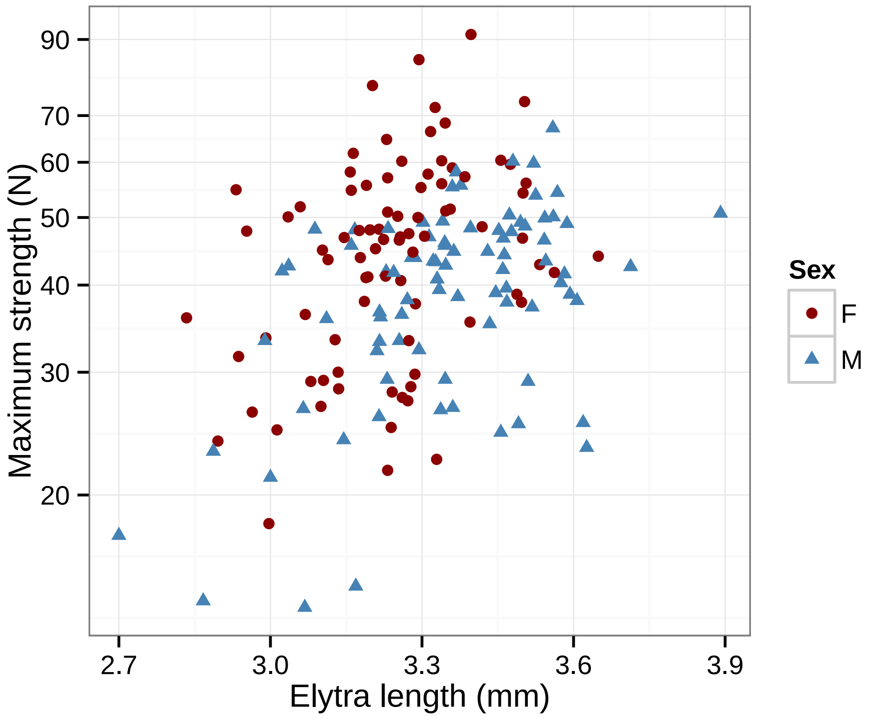

Figure 2:

Figure B: Log maximum strength plotted against elytra length for male and female beetles.

```
# Comparison of size

t.test(Elytra ~ Sex, var.equal = TRUE)
```

```
##
##  Two Sample t-test
##
## data:  Elytra by Sex
## t = -3.4842, df = 185, p-value = 0.0006162
## alternative hypothesis: true difference in means is not equal to 0
## 95 percent confidence interval:
##  -0.14868766 -0.04117847
```

```
## sample estimates:
## mean in group F mean in group M
##        3.240120        3.335053
```

```r
# Comparison of strength

strengthmod1 <- lm(log(MaxPerform) ~ Sex * Elytra)
## Fit initial model
drop1(strengthmod1, sex = "F")
```

```
## Single term deletions
##
## Model:
## log(MaxPerform) ~ Sex * Elytra
##            Df Sum of Sq    RSS     AIC
## <none>                  14.558 -402.90
## Sex:Elytra  1  0.001018 14.559 -404.89
```

```r
strengthmod2 <- update(strengthmod1, ~. - Sex:Elytra)
## No significant interaction so refit model only main effects

drop1(strengthmod2, test = "F")
```

```
## Single term deletions
##
## Model:
## log(MaxPerform) ~ Sex + Elytra
##        Df Sum of Sq    RSS     AIC F value    Pr(>F)
## <none>              14.559 -404.89
## Sex     1    1.8125 16.371 -387.18  20.542 1.116e-05 ***
## Elytra  1    3.4897 18.049 -370.79  39.550 2.758e-09 ***
## ---
## Signif. codes:  0 '***' 0.001 '**' 0.01 '*' 0.05 '.' 0.1 ' ' 1
```

```r
detach(Beetle)

## Both main effects highly signficant

summary(strengthmod2)
```

```
##
## Call:
## lm(formula = log(MaxPerform) ~ Sex + Elytra)
##
## Residuals:
##      Min       1Q   Median       3Q      Max
## -0.80386 -0.17680  0.06066  0.18730  0.62068
##
## Coefficients:
##             Estimate Std. Error t value Pr(>|t|)
## (Intercept)   1.2057     0.4095   2.944   0.0037 **
## SexM         -0.2166     0.0478  -4.532 1.12e-05 ***
```

```
## Elytra        0.7919     0.1259   6.289 2.76e-09 ***
## ---
## Signif. codes:  0 '***' 0.001 '**' 0.01 '*' 0.05 '.' 0.1 ' ' 1
##
## Residual standard error: 0.297 on 165 degrees of freedom
##   (23 observations deleted due to missingness)
## Multiple R-squared:  0.2245, Adjusted R-squared:  0.2151
## F-statistic: 23.88 on 2 and 165 DF,  p-value: 7.763e-10
```

```r
rm(Beetle)
```

4 Path analysis for male performance

```
# setwd('~/Dropbox/Current/Leeann's resource
# availablity/Analysis 2014/path analysis 2014')'
library(lavaan)
```

```
## Warning: package 'lavaan' was built under R version 3.1.3
```

```
## This is lavaan 0.5-18
## lavaan is BETA software! Please report any bugs.
```

```
maledata <- read.table("maledata.txt")
```

4.1 Data transformations used:

Haemolymph protein and maximum strength were both log transformed to reduce heteroscedasticity Weight gain was log transformed to reduce curvature in the relationship between weight gain and maximum strength Eclosion weight and fat content were rescaled by dividing by 100 in order to make the scales the variables are measured on more equivalent.

4.2 Initial model

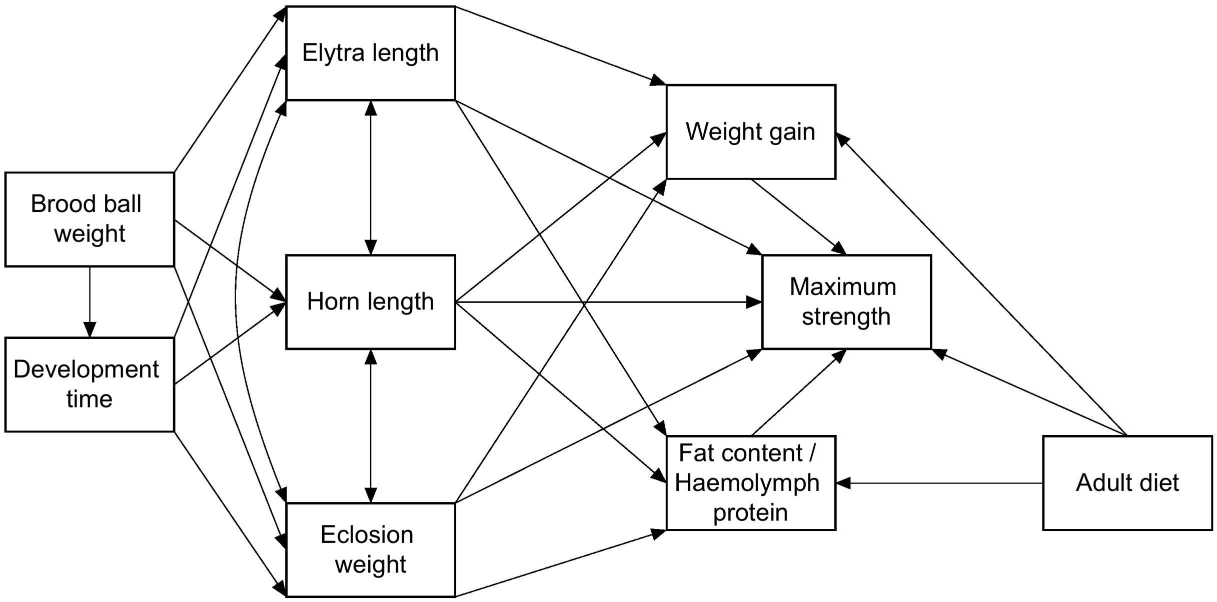

Figure C: Path diagram showing the original model fitted to explain male performance. Double headed arrows indicate correlation (i.e. no assumptions made about causality

```
##Specify the model

Path.full.model.perform.male<-'#Regressions
```

```
lnPerform~lnProt+Fat.trans+lnWtgain+Elytra+Horn+Ec.Weight.trans+Adult.diet
lnProt~Elytra+Ec.Weight.trans+lnWtgain+Horn+Adult.diet
Fat.trans~Elytra+Ec.Weight.trans+lnWtgain+Horn+Adult.diet
lnWtgain~Elytra+Ec.Weight.trans+EndBBweight+Horn+Adult.diet
Ec.Weight.trans~EndBBweight +Developmenttime
Elytra~EndBBweight +Developmenttime
Developmenttime~EndBBweight
Horn~ EndBBweight+Developmenttime

#covariances
Horn~~Elytra
Horn~~Ec.Weight.trans
lnProt~~Fat.trans
Elytra~~Ec.Weight.trans
'
```

```
## Fit the model and check the summary

Male.performance.full.model <- sem(Path.full.model.perform.male,
    data = maledata, missing = "fiml")
summary(Male.performance.full.model, standardized = TRUE)
```

```
## lavaan (0.5-18) converged normally after 156 iterations
##
##   Number of observations                            98
##
##   Number of missing patterns                         8
##
##   Estimator                                         ML
##   Minimum Function Test Statistic               13.481
##   Degrees of freedom                                11
##   P-value (Chi-square)                           0.263
##
## Parameter estimates:
##
##   Information                                 Observed
##   Standard Errors                             Standard
##
##                    Estimate  Std.err  Z-value  P(>|z|)   Std.lv  Std.all
## Regressions:
##   lnPerform ~
##     lnProt           -0.006    0.068   -0.094    0.925   -0.006   -0.008
##     Fat.trans         0.102    0.245    0.418    0.676    0.102    0.054
##     lnWtgain          0.543    0.280    1.938    0.053    0.543    0.385
##     Elytra            0.281    0.264    1.063    0.288    0.281    0.168
##     Horn              0.647    0.378    1.714    0.086    0.647    0.377
##     Ec.Weght.trns    -0.163    0.088   -1.862    0.063   -0.163   -0.300
##     Adult.diet        0.009    0.069    0.130    0.897    0.009    0.012
##   lnProt ~
##     Elytra           -0.750    0.463   -1.620    0.105   -0.750   -0.353
##     Ec.Weght.trns     0.114    0.146    0.781    0.435    0.114    0.165
##     lnWtgain         -0.028    0.418   -0.067    0.946   -0.028   -0.016
##     Horn              0.157    0.670    0.234    0.815    0.157    0.072
```

```
##     Adult.diet        0.087    0.099    0.871    0.384    0.087    0.095
##   Fat.trans ~
##     Elytra            0.050    0.127    0.393    0.694    0.050    0.057
##     Ec.Weght.trns     0.031    0.040    0.776    0.438    0.031    0.108
##     lnWtgain          0.527    0.109    4.830    0.000    0.527    0.714
##     Horn             -0.224    0.179   -1.252    0.211   -0.224   -0.250
##     Adult.diet       -0.164    0.027   -6.061    0.000   -0.164   -0.438
##   lnWtgain ~
##     Elytra            0.353    0.120    2.939    0.003    0.353    0.298
##     Ec.Weght.trns    -0.095    0.041   -2.315    0.021   -0.095   -0.246
##     EndBBweight      -0.027    0.045   -0.610    0.542   -0.027   -0.041
##     Horn              1.015    0.137    7.395    0.000    1.015    0.834
##     Adult.diet       -0.008    0.027   -0.281    0.779   -0.008   -0.015
##   Ec.Weight.trans ~
##     EndBBweight       1.109    0.136    8.145    0.000    1.109    0.641
##     Developmenttm    -0.019    0.020   -0.938    0.348   -0.019   -0.074
##   Elytra ~
##     EndBBweight       0.337    0.046    7.333    0.000    0.337    0.599
##     Developmenttm    -0.019    0.007   -2.783    0.005   -0.019   -0.230
##   Developmenttime ~
##     EndBBweight       0.895    0.679    1.319    0.187    0.895    0.132
##   Horn ~
##     EndBBweight       0.305    0.045    6.811    0.000    0.305    0.557
##     Developmenttm    -0.026    0.007   -3.952    0.000   -0.026   -0.327
##
## Covariances:
##   Elytra ~~
##     Horn              0.021    0.004    5.740    0.000    0.021    0.740
##   Ec.Weight.trans ~~
##     Horn              0.065    0.011    6.021    0.000    0.065    0.781
##   lnProt ~~
##     Fat.trans        -0.011    0.006   -1.787    0.074   -0.011   -0.208
##   Ec.Weight.trans ~~
##     Elytra            0.059    0.011    5.497    0.000    0.059    0.686
##
## Intercepts:
##     lnPerform        -0.592    1.400   -0.423    0.672   -0.592   -1.658
##     lnProt           10.255    2.008    5.106    0.000   10.255   22.591
##     Fat.trans        -2.356    0.533   -4.420    0.000   -2.356  -12.620
##     lnWtgain          3.970    0.295   13.464    0.000    3.970   15.688
##     Ec.Weght.trns     2.574    0.621    4.144    0.000    2.574    3.918
##     Elytra            3.419    0.212   16.153    0.000    3.419   16.021
##     Developmenttm    29.224    1.016   28.751    0.000   29.224   11.361
##     Horn              1.412    0.206    6.850    0.000    1.412    6.785
##
## Variances:
##     lnPerform         0.065    0.010                      0.065    0.511
##     lnProt            0.195    0.031                      0.195    0.947
##     Fat.trans         0.015    0.002                      0.015    0.429
##     lnWtgain          0.015    0.002                      0.015    0.234
##     Ec.Weght.trns     0.257    0.037                      0.257    0.596
##     Elytra            0.028    0.004                      0.028    0.625
##     Developmenttm     6.502    0.929                      6.502    0.983
##     Horn              0.027    0.004                      0.027    0.631
```

4.3 Final model

A final model can be produced by removing non-significant terms, adding in ones indicated by modification indices and by removing terms which have no direct or indirect effect on performance. Mdification indices are used for model refinement: they give an index of the change in goodness of fit from adding a term into a model. The final path model for male performance is:

```
Path.reduced.model.perform.male<-'#Regressions


lnPerform~lnWtgain+Elytra+EndBBweight
lnWtgain~Elytra+Ec.Weight.trans+Horn
Ec.Weight.trans~EndBBweight
Elytra~EndBBweight+Developmenttime
Horn~ EndBBweight+Developmenttime

#covariances
Horn~~Ec.Weight.trans
Elytra~~Ec.Weight.trans
Elytra~~Horn
'
```

```
Male.performance.reduced.model <- sem(Path.reduced.model.perform.male,
    data = maledata, missing = "fiml")
summary(Male.performance.reduced.model, standardized = TRUE,
    fit.measures = TRUE)
```

```
## lavaan (0.5-18) converged normally after  91 iterations
##
##   Number of observations                            98
##
##   Number of missing patterns                         4
##
##   Estimator                                         ML
##   Minimum Function Test Statistic                4.931
##   Degrees of freedom                                 6
##   P-value (Chi-square)                           0.553
##
## Model test baseline model:
##
##   Minimum Function Test Statistic              453.425
##   Degrees of freedom                                20
##   P-value                                        0.000
##
## User model versus baseline model:
##
##   Comparative Fit Index (CFI)                    1.000
##   Tucker-Lewis Index (TLI)                       1.008
##
## Loglikelihood and Information Criteria:
##
##   Loglikelihood user model (H0)               -137.508
##   Loglikelihood unrestricted model (H1)       -135.042
##
##   Number of free parameters                         24
```

```
##   Akaike (AIC)                                 323.016
##   Bayesian (BIC)                               385.055
##   Sample-size adjusted Bayesian (BIC)          309.266
##
## Root Mean Square Error of Approximation:
##
##   RMSEA                                          0.000
##   90 Percent Confidence Interval          0.000  0.118
##   P-value RMSEA <= 0.05                          0.688
##
## Standardized Root Mean Square Residual:
##
##   SRMR                                           0.036
##
## Parameter estimates:
##
##   Information                                 Observed
##   Standard Errors                             Standard
##
##                    Estimate  Std.err  Z-value  P(>|z|)   Std.lv  Std.all
## Regressions:
##   lnPerform ~
##     lnWtgain          0.770    0.201    3.826    0.000    0.770    0.551
##     Elytra            0.455    0.227    2.007    0.045    0.455    0.275
##     EndBBweight      -0.221    0.087   -2.547    0.011   -0.221   -0.241
##   lnWtgain ~
##     Elytra            0.343    0.118    2.896    0.004    0.343    0.290
##     Ec.Weght.trns    -0.105    0.038   -2.781    0.005   -0.105   -0.277
##     Horn              1.025    0.137    7.500    0.000    1.025    0.839
##   Ec.Weight.trans ~
##     EndBBweight       1.092    0.136    8.056    0.000    1.092    0.631
##   Elytra ~
##     EndBBweight       0.333    0.046    7.266    0.000    0.333    0.598
##     Developmenttm    -0.015    0.005   -2.902    0.004   -0.015   -0.180
##   Horn ~
##     EndBBweight       0.301    0.045    6.734    0.000    0.301    0.558
##     Developmenttm    -0.022    0.004   -5.039    0.000   -0.022   -0.271
##
## Covariances:
##   Ec.Weight.trans ~~
##     Horn              0.066    0.011    6.021    0.000    0.066    0.783
##     Elytra            0.059    0.011    5.497    0.000    0.059    0.687
##   Elytra ~~
##     Horn              0.021    0.004    5.734    0.000    0.021    0.742
##
## Intercepts:
##     lnPerform        -2.061    0.830   -2.483    0.013   -2.061   -5.897
##     lnWtgain          3.980    0.286   13.909    0.000    3.980   15.905
##     Ec.Weght.trns     2.023    0.203    9.960    0.000    2.023    3.080
##     Elytra            3.294    0.164   20.091    0.000    3.294   15.575
##     Horn              1.272    0.142    8.962    0.000    1.272    6.209
##
## Variances:
##     lnPerform         0.064    0.010                      0.064    0.520
```

```
##      lnWtgain           0.015    0.002                       0.015    0.241
##      Ec.Weght.trns      0.259    0.037                       0.259    0.602
##      Elytra             0.029    0.004                       0.029    0.639
##      Horn               0.027    0.004                       0.027    0.655
```

4.4 Notes

- The "std.all" column gives the coefficient estimates when all predictors are standardised, meaning you can make meaningful comparisons between them (i.e. horn length has a bigger influence on weight gain than elytra length)
- Diet drops out of the path model so there's no problem with the binomial predictor variables in this case.
- In the fit measures section, the first p-value under "Minimum Function Chi Square" should be close to 1 for a well fitted model. In this case it is.
- RMSEA values close to zero are indicative of a well fitted model, again this meets the criterion.
- Brood ball weight added as a predictor for performance after checking modification indices.

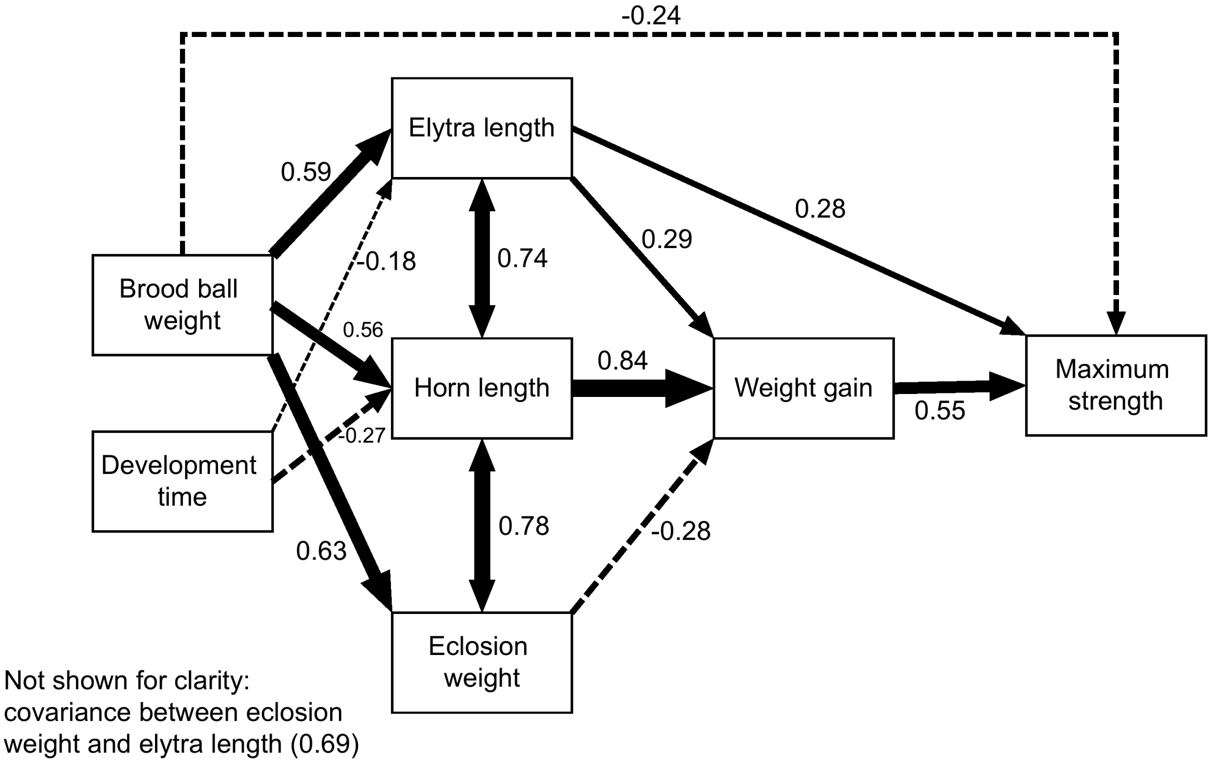

Figure D: Path diagram for the final model explaining male performance. Solid lines are positive relations, dashed lines negative, double headed arrows indicate correlation (i.e. no assumptions about causality), line width is proportional to the strength of the relationship.

5 Path analysis for female performance

5.1 Data transformations used:

The same transformations were used as for the analysis of male performance:

Haemolymph protein and maximum strength were both log transformed to reduce heteroscedasticity Weight gain was log transformed to reduce curvature in the relationship between weight gain and maximum strength Eclosion weight and fat content were rescaled by dividing by 100 in order to make the scales the variables are measured on more equivalent.

5.2 Initial model

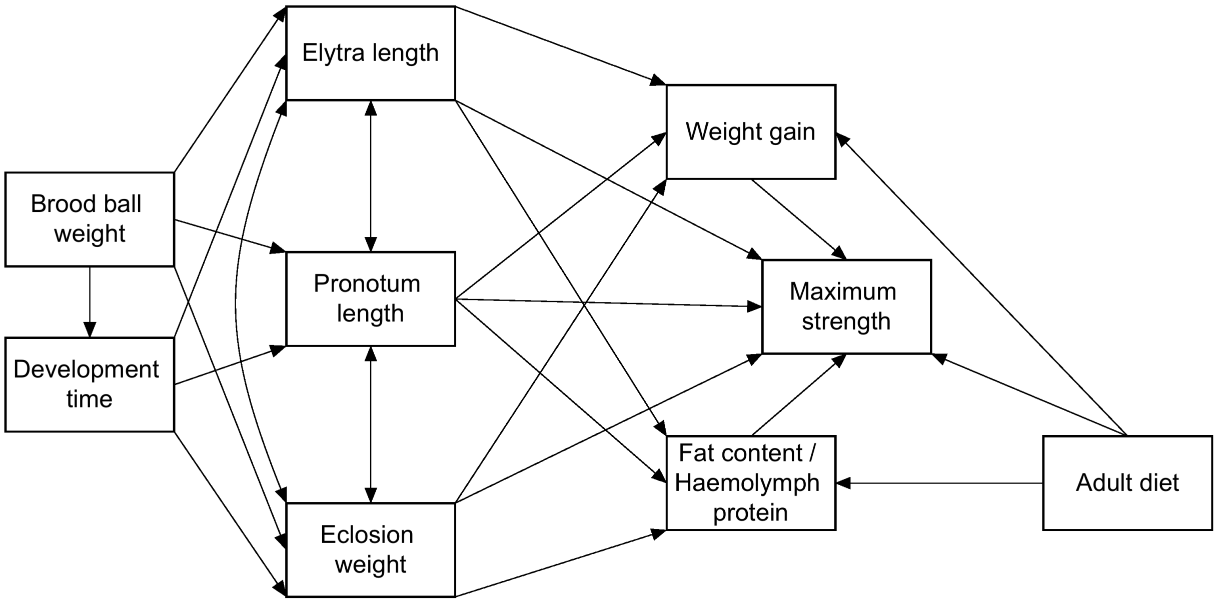

Figure E: Path diagram showing the original model fitted to explain female performance. Double headed arrows indicate correlation (i.e. no assumptions made about causality)

```
#Load data
femaledata<-read.table("femaledata.txt",header=TRUE)

#Specify full model
Path.full.model.perform.female<-'#Regressions


lnPerform~lnProt+Fat.trans+lnWtgain+Elytra+Ec.Weight.trans+Adult.diet
lnProt~Elytra+Ec.Weight.trans+lnWtgain+Adult.diet
Fat.trans~Elytra+Ec.Weight.trans+lnWtgain +Adult.diet
lnWtgain~Elytra+Ec.Weight.trans+EndBBweight +Adult.diet
Ec.Weight.trans~EndBBweight +Developmenttime
Elytra~EndBBweight +Developmenttime
Developmenttime~EndBBweight

#covariances
lnProt~~Fat.trans
Elytra~~Ec.Weight.trans'
```

```
## Fit the model and check the summary

Female.performance.full.model <- sem(Path.full.model.perform.female,
    data = femaledata, missing = "fiml")
summary(Female.performance.full.model, standardized = TRUE)
```

```
## lavaan (0.5-18) converged normally after 135 iterations
##
##   Number of observations                            93
##
##   Number of missing patterns                         5
##
##   Estimator                                         ML
##   Minimum Function Test Statistic               12.009
##   Degrees of freedom                                10
##   P-value (Chi-square)                           0.284
##
## Parameter estimates:
##
##   Information                                 Observed
##   Standard Errors                             Standard
##
##                    Estimate  Std.err  Z-value  P(>|z|)   Std.lv  Std.all
## Regressions:
##   lnPerform ~
##     lnProt           -0.032    0.105   -0.307    0.759   -0.032   -0.032
##     Fat.trans         0.148    0.109    1.362    0.173    0.148    0.200
##     lnWtgain          0.107    0.166    0.644    0.519    0.107    0.097
##     Elytra            0.010    0.151    0.063    0.950    0.010    0.011
##     Ec.Weght.trns     0.083    0.052    1.605    0.109    0.083    0.262
##     Adult.diet        0.026    0.034    0.780    0.436    0.026    0.091
##   lnProt ~
##     Elytra           -0.305    0.153   -1.998    0.046   -0.305   -0.342
##     Ec.Weght.trns     0.127    0.050    2.514    0.012    0.127    0.397
##     lnWtgain          0.223    0.151    1.470    0.142    0.223    0.203
##     Adult.diet        0.062    0.029    2.128    0.033    0.062    0.216
##   Fat.trans ~
##     Elytra            0.031    0.149    0.207    0.836    0.031    0.026
##     Ec.Weght.trns     0.099    0.049    2.015    0.044    0.099    0.232
##     lnWtgain          0.661    0.147    4.499    0.000    0.661    0.448
##     Adult.diet       -0.158    0.029   -5.501    0.000   -0.158   -0.406
##   lnWtgain ~
##     Elytra            0.396    0.101    3.922    0.000    0.396    0.487
##     Ec.Weght.trns     0.079    0.037    2.125    0.034    0.079    0.272
##     EndBBweight      -0.012    0.035   -0.339    0.735   -0.012   -0.031
##     Adult.diet        0.000    0.021    0.014    0.989    0.000    0.001
##   Ec.Weight.trans ~
##     EndBBweight       0.641    0.118    5.410    0.000    0.641    0.485
##     Developmenttm    -0.020    0.013   -1.476    0.140   -0.020   -0.132
##   Elytra ~
##     EndBBweight       0.184    0.044    4.222    0.000    0.184    0.389
##     Developmenttm    -0.012    0.005   -2.436    0.015   -0.012   -0.225
##   Developmenttime ~
##     EndBBweight      -1.432    0.917   -1.563    0.118   -1.432   -0.160
##
## Covariances:
##   lnProt ~~
##     Fat.trans        -0.001    0.002   -0.727    0.467   -0.001   -0.079
##   Ec.Weight.trans ~~
##     Elytra            0.036    0.007    5.308    0.000    0.036    0.659
```

```
##
## Intercepts:
##     lnPerform         0.722    0.840    0.860    0.390    0.722    5.033
##     lnProt            3.044    0.720    4.230    0.000    3.044   21.219
##     Fat.trans        -3.328    0.698   -4.765    0.000   -3.328  -17.250
##     lnWtgain          4.167    0.247   16.874    0.000    4.167   31.901
##     Ec.Weght.trns     2.940    0.448    6.557    0.000    2.940    6.537
##     Elytra            3.335    0.166   20.098    0.000    3.335   20.734
##     Developmenttm    31.446    1.314   23.936    0.000   31.446   10.323
##
## Variances:
##     lnPerform         0.016    0.002                      0.016    0.777
##     lnProt            0.017    0.003                      0.017    0.831
##     Fat.trans         0.016    0.002                      0.016    0.433
##     lnWtgain          0.009    0.001                      0.009    0.513
##     Ec.Weght.trns     0.147    0.022                      0.147    0.727
##     Elytra            0.020    0.003                      0.020    0.769
##     Developmenttm     9.042    1.326                      9.042    0.974
```

5.3 Final model

Model reduction leads to this:

```
#Specify reduced model
Path.reduced.model.perform.female<-'#Regressions

lnPerform~Ec.Weight.trans
Ec.Weight.trans~EndBBweight'
```

```
# Fit the model and check the summary

female.performance.reduced.model <- sem(Path.reduced.model.perform.female,
    data = femaledata, missing = "fiml")
summary(female.performance.reduced.model, standardized = TRUE,
    fit.measures = TRUE)
```

```
## lavaan (0.5-18) converged normally after  25 iterations
##
##   Number of observations                            93
##
##   Number of missing patterns                         2
##
##   Estimator                                         ML
##   Minimum Function Test Statistic                1.716
##   Degrees of freedom                                 1
##   P-value (Chi-square)                           0.190
##
## Model test baseline model:
##
##   Minimum Function Test Statistic               46.424
##   Degrees of freedom                                 3
##   P-value                                        0.000
##
```

```
## User model versus baseline model:
##
##   Comparative Fit Index (CFI)                    0.984
##   Tucker-Lewis Index (TLI)                       0.951
##
## Loglikelihood and Information Criteria:
##
##   Loglikelihood user model (H0)                -23.327
##   Loglikelihood unrestricted model (H1)        -22.469
##
##   Number of free parameters                          6
##   Akaike (AIC)                                  58.653
##   Bayesian (BIC)                                73.849
##   Sample-size adjusted Bayesian (BIC)           54.908
##
## Root Mean Square Error of Approximation:
##
##   RMSEA                                          0.088
##   90 Percent Confidence Interval          0.000  0.306
##   P-value RMSEA <= 0.05                          0.241
##
## Standardized Root Mean Square Residual:
##
##   SRMR                                           0.036
##
## Parameter estimates:
##
##   Information                                 Observed
##   Standard Errors                             Standard
##
##                    Estimate  Std.err  Z-value  P(>|z|)   Std.lv  Std.all
## Regressions:
##   lnPerform ~
##     Ec.Weght.trns     0.136    0.031    4.373    0.000    0.136    0.427
##   Ec.Weight.trans ~
##     EndBBweight       0.669    0.118    5.654    0.000    0.669    0.506
##
## Intercepts:
##     lnPerform         1.197    0.102   11.733    0.000    1.197    8.334
##     Ec.Weght.trns     2.327    0.170   13.725    0.000    2.327    5.173
##
## Variances:
##     lnPerform         0.017    0.003                      0.017    0.818
##     Ec.Weght.trns     0.151    0.022                      0.151    0.744
```

This model is not as good a fit as the one for males but it is acceptable.

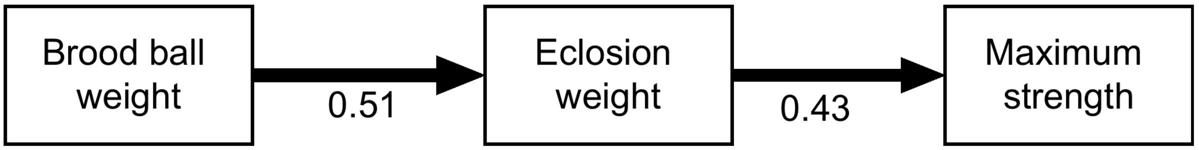

Figure F: Path diagram for the final model explaining female performance. Solid lines are positive relations, line width is

proportional to the strength of the relationship.

6 Path analysis for male testes mass and fat content

6.1 Initial model

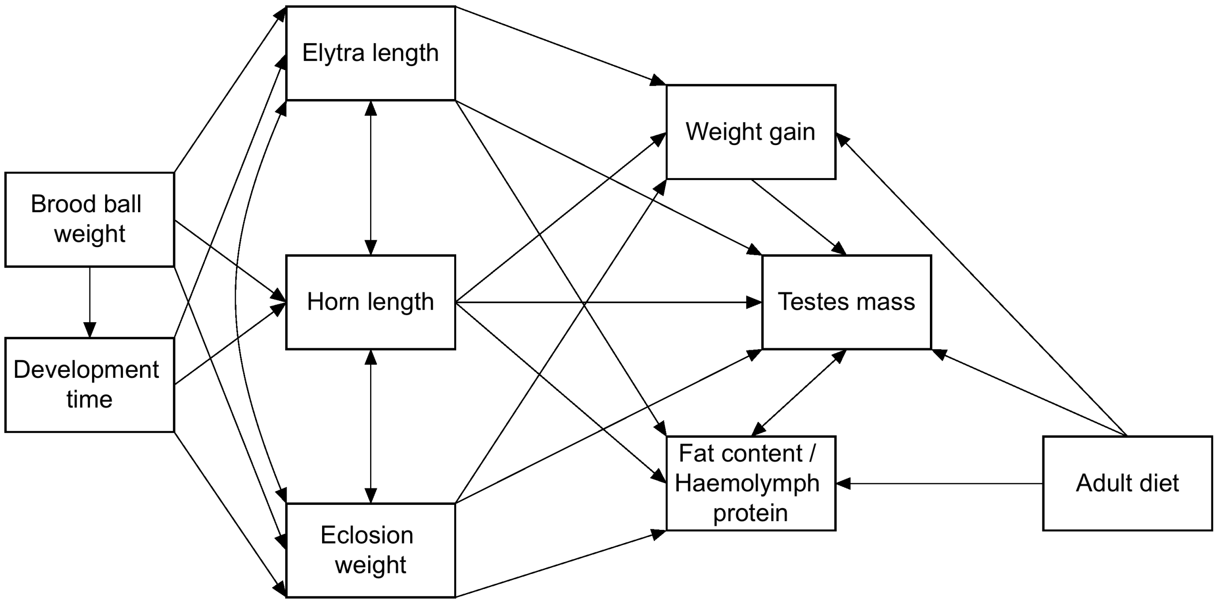

Figure G: Path diagram showing the original model fitted to explain testes mass. Double headed arrows indicate correlation (i.e. no assumptions made about causality)

```
#Specify full model

Path.full.model.testes<-'#Regressions



 lnTestes~Elytra+Ec.Weight.trans+lnWtgain+Horn+Adult.diet
 lnProt~Elytra+Ec.Weight.trans+lnWtgain+Horn+Adult.diet
 Fat.trans~Elytra+Ec.Weight.trans+lnWtgain+Horn+Adult.diet
 lnWtgain~Elytra+Ec.Weight.trans+EndBBweight+Horn+Adult.diet
 Ec.Weight.trans~EndBBweight +Developmenttime
 Elytra~EndBBweight +Developmenttime
 Developmenttime~EndBBweight
 Horn~ EndBBweight+Developmenttime

 #covariances
 lnTestes~~Fat.trans
 Elytra~~Ec.Weight.trans
 Horn~~Elytra
 Horn~~Ec.Weight.trans'
```

```r
# Fit the model and check the summary


male.testes.full.model <- sem(Path.full.model.testes, data = maledata,
    missing = "fiml")
summary(male.testes.full.model, standardized = TRUE, fit.measures = TRUE)
```

```
## lavaan (0.5-18) converged normally after 170 iterations
##
##   Number of observations                            98
##
##   Number of missing patterns                         8
##
##   Estimator                                         ML
##   Minimum Function Test Statistic               11.983
##   Degrees of freedom                                11
##   P-value (Chi-square)                           0.365
##
## Model test baseline model:
##
##   Minimum Function Test Statistic              525.548
##   Degrees of freedom                                44
##   P-value                                        0.000
##
## User model versus baseline model:
##
##   Comparative Fit Index (CFI)                    0.998
##   Tucker-Lewis Index (TLI)                       0.992
##
## Loglikelihood and Information Criteria:
##
##   Loglikelihood user model (H0)               -174.026
##   Loglikelihood unrestricted model (H1)       -168.035
##
##   Number of free parameters                         49
##   Akaike (AIC)                                 446.052
##   Bayesian (BIC)                               572.716
##   Sample-size adjusted Bayesian (BIC)          417.981
##
## Root Mean Square Error of Approximation:
##
##   RMSEA                                          0.030
##   90 Percent Confidence Interval          0.000  0.113
##   P-value RMSEA <= 0.05                          0.569
##
## Standardized Root Mean Square Residual:
##
##   SRMR                                           0.041
##
## Parameter estimates:
##
##   Information                                 Observed
##   Standard Errors                             Standard
```

```
##
##                   Estimate  Std.err  Z-value  P(>|z|)   Std.lv  Std.all
## Regressions:
##   lnTestes ~
##     Elytra           0.136    0.202    0.673    0.501    0.136    0.129
##     Ec.Weght.trns    0.073    0.064    1.140    0.254    0.073    0.212
##     lnWtgain         0.457    0.176    2.596    0.009    0.457    0.515
##     Horn            -0.412    0.291   -1.416    0.157   -0.412   -0.382
##     Adult.diet      -0.073    0.043   -1.688    0.091   -0.073   -0.163
##   lnProt ~
##     Elytra          -0.748    0.463   -1.617    0.106   -0.748   -0.352
##     Ec.Weght.trns    0.114    0.146    0.779    0.436    0.114    0.165
##     lnWtgain        -0.030    0.417   -0.072    0.942   -0.030   -0.017
##     Horn             0.160    0.668    0.240    0.810    0.160    0.073
##     Adult.diet       0.088    0.099    0.889    0.374    0.088    0.097
##   Fat.trans ~
##     Elytra           0.057    0.126    0.448    0.654    0.057    0.065
##     Ec.Weght.trns    0.030    0.040    0.761    0.447    0.030    0.106
##     lnWtgain         0.529    0.109    4.852    0.000    0.529    0.715
##     Horn            -0.230    0.179   -1.285    0.199   -0.230   -0.256
##     Adult.diet      -0.165    0.027   -6.100    0.000   -0.165   -0.439
##   lnWtgain ~
##     Elytra           0.353    0.120    2.938    0.003    0.353    0.298
##     Ec.Weght.trns   -0.095    0.041   -2.315    0.021   -0.095   -0.246
##     EndBBweight     -0.027    0.045   -0.610    0.542   -0.027   -0.041
##     Horn             1.015    0.137    7.396    0.000    1.015    0.834
##     Adult.diet      -0.008    0.027   -0.280    0.779   -0.008   -0.015
##   Ec.Weight.trans ~
##     EndBBweight      1.109    0.136    8.145    0.000    1.109    0.641
##     Developmenttm   -0.019    0.020   -0.938    0.348   -0.019   -0.074
##   Elytra ~
##     EndBBweight      0.337    0.046    7.333    0.000    0.337    0.599
##     Developmenttm   -0.019    0.007   -2.783    0.005   -0.019   -0.230
##   Developmenttime ~
##     EndBBweight      0.895    0.679    1.319    0.187    0.895    0.132
##   Horn ~
##     EndBBweight      0.305    0.045    6.811    0.000    0.305    0.557
##     Developmenttm   -0.026    0.007   -3.952    0.000   -0.026   -0.327
##
## Covariances:
##   lnTestes ~~
##     Fat.trans        0.006    0.003    2.211    0.027    0.006    0.251
##   Ec.Weight.trans ~~
##     Elytra           0.059    0.011    5.497    0.000    0.059    0.686
##   Elytra ~~
##     Horn             0.021    0.004    5.740    0.000    0.021    0.740
##   Ec.Weight.trans ~~
##     Horn             0.065    0.011    6.021    0.000    0.065    0.781
##   lnTestes ~~
##     lnProt          -0.001    0.010   -0.112    0.911   -0.001   -0.013
##   lnProt ~~
##     Fat.trans       -0.011    0.006   -1.781    0.075   -0.011   -0.207
##
## Intercepts:
```

```
##     lnTestes          0.087    0.858    0.101    0.919    0.087    0.388
##     lnProt           10.256    2.001    5.125    0.000   10.256   22.592
##     Fat.trans        -2.380    0.532   -4.469    0.000   -2.380  -12.721
##     lnWtgain          3.970    0.295   13.465    0.000    3.970   15.688
##     Ec.Weght.trns     2.574    0.621    4.144    0.000    2.574    3.918
##     Elytra            3.419    0.212   16.153    0.000    3.419   16.021
##     Developmenttm    29.224    1.016   28.751    0.000   29.224   11.361
##     Horn              1.412    0.206    6.850    0.000    1.412    6.785
##
## Variances:
##     lnTestes          0.039    0.006                      0.039    0.768
##     lnProt            0.195    0.031                      0.195    0.946
##     Fat.trans         0.015    0.002                      0.015    0.427
##     lnWtgain          0.015    0.002                      0.015    0.234
##     Ec.Weght.trns     0.257    0.037                      0.257    0.596
##     Elytra            0.028    0.004                      0.028    0.625
##     Developmenttm     6.502    0.929                      6.502    0.983
##     Horn              0.027    0.004                      0.027    0.631
```

6.2 Final model

```
#Specify reduced model

Path.reduced.model.testes<-'#Regressions


lnTestes~lnWtgain+Developmenttime
Fat.trans~lnWtgain+Adult.diet
lnWtgain~Elytra+Ec.Weight.trans+Horn
Ec.Weight.trans~EndBBweight
Elytra~EndBBweight +Developmenttime
Horn~ EndBBweight+Developmenttime

 #covariances
lnTestes~~Fat.trans
Elytra~~Ec.Weight.trans
Horn~~Elytra
Horn~~Ec.Weight.trans'
```

```
# Fit the model and check the summary

male.testes.reduced.model <- sem(Path.reduced.model.testes, data = maledata,
    missing = "fiml")
summary(male.testes.reduced.model, standardized = TRUE, fit.measures = TRUE)
```

```
## lavaan (0.5-18) converged normally after 116 iterations
##
##   Number of observations                            98
##
##   Number of missing patterns                         7
##
```

```
##   Estimator                                         ML
##   Minimum Function Test Statistic               11.606
##   Degrees of freedom                                17
##   P-value (Chi-square)                           0.823
##
## Model test baseline model:
##
##   Minimum Function Test Statistic              513.155
##   Degrees of freedom                                33
##   P-value                                        0.000
##
## User model versus baseline model:
##
##   Comparative Fit Index (CFI)                    1.000
##   Tucker-Lewis Index (TLI)                       1.022
##
## Loglikelihood and Information Criteria:
##
##   Loglikelihood user model (H0)               -128.717
##   Loglikelihood unrestricted model (H1)       -122.914
##
##   Number of free parameters                         28
##   Akaike (AIC)                                 313.434
##   Bayesian (BIC)                               385.813
##   Sample-size adjusted Bayesian (BIC)          297.393
##
## Root Mean Square Error of Approximation:
##
##   RMSEA                                          0.000
##   90 Percent Confidence Interval          0.000  0.057
##   P-value RMSEA <= 0.05                          0.933
##
## Standardized Root Mean Square Residual:
##
##   SRMR                                           0.057
##
## Parameter estimates:
##
##   Information                                 Observed
##   Standard Errors                             Standard
##
##                    Estimate  Std.err  Z-value  P(>|z|)   Std.lv  Std.all
## Regressions:
##   lnTestes ~
##     lnWtgain          0.445    0.093    4.781    0.000    0.445    0.492
##     Developmenttm     0.019    0.008    2.354    0.019    0.019    0.222
##   Fat.trans ~
##     lnWtgain          0.459    0.057    8.085    0.000    0.459    0.626
##     Adult.diet       -0.155    0.027   -5.830    0.000   -0.155   -0.421
##   lnWtgain ~
##     Elytra            0.342    0.118    2.893    0.004    0.342    0.289
##     Ec.Weght.trns    -0.105    0.038   -2.782    0.005   -0.105   -0.277
##     Horn              1.026    0.136    7.522    0.000    1.026    0.840
##   Ec.Weight.trans ~
```

```
##     EndBBweight       1.092    0.136    8.056    0.000    1.092    0.631
##   Elytra ~
##     EndBBweight       0.333    0.046    7.266    0.000    0.333    0.598
##     Developmenttm    -0.015    0.005   -2.902    0.004   -0.015   -0.180
##   Horn ~
##     EndBBweight       0.301    0.045    6.734    0.000    0.301    0.558
##     Developmenttm    -0.022    0.004   -5.039    0.000   -0.022   -0.271
##
## Covariances:
##   lnTestes ~~
##     Fat.trans         0.007    0.003    2.352    0.019    0.007    0.272
##   Ec.Weight.trans ~~
##     Elytra            0.059    0.011    5.497    0.000    0.059    0.687
##   Elytra ~~
##     Horn              0.021    0.004    5.734    0.000    0.021    0.742
##   Ec.Weight.trans ~~
##     Horn              0.066    0.011    6.021    0.000    0.066    0.783
##
## Intercepts:
##     lnTestes         -0.269    0.654   -0.411    0.681   -0.269   -1.190
##     Fat.trans        -1.935    0.336   -5.766    0.000   -1.935  -10.540
##     lnWtgain          3.982    0.286   13.938    0.000    3.982   15.913
##     Ec.Weght.trns     2.023    0.203    9.960    0.000    2.023    3.080
##     Elytra            3.294    0.164   20.091    0.000    3.294   15.575
##     Horn              1.272    0.142    8.962    0.000    1.272    6.209
##
## Variances:
##     lnTestes          0.039    0.006                      0.039    0.756
##     Fat.trans         0.015    0.002                      0.015    0.454
##     lnWtgain          0.015    0.002                      0.015    0.241
##     Ec.Weght.trns     0.259    0.037                      0.259    0.602
##     Elytra            0.029    0.004                      0.029    0.639
##     Horn              0.027    0.004                      0.027    0.655
```

Once again this is a well-fitted model. NB development time has been added in as a direct predictor of testes mass, this was a consequence of inspecting modification indices. The final path model looks like this:

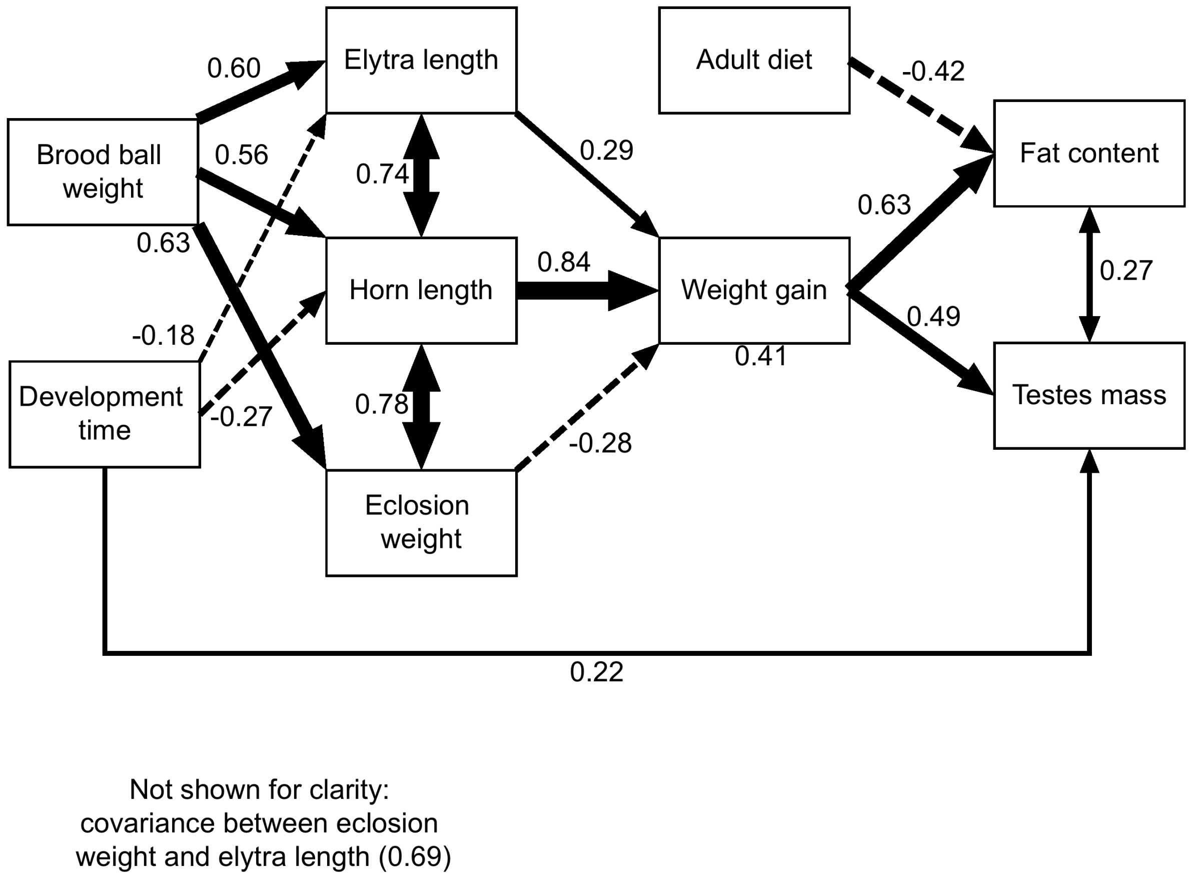

Figure H: Path diagram for the final model explaining testes mass. Solid lines are positive relations, dashed lines negative, double headed arrows indicate correlation (i.e. no assumptions about causality), line width is proportional to the strength of the relationship.

7 Path analysis for female fat content

7.1 Initial model

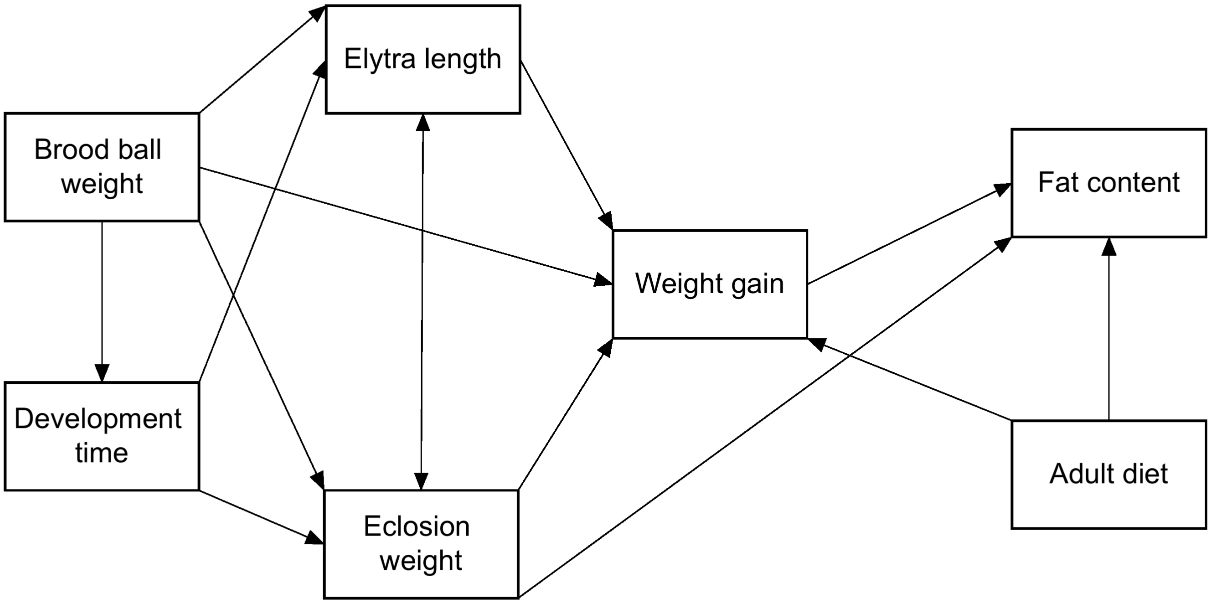

Figure I: Path diagram showing the original model fitted to explain fat content in females. Double headed arrows indicate correlation (i.e. no assumptions made about causality)

```
#Specify the full model


Path.full.model.fat<-'#Regressions

lnProt~Elytra+Ec.Weight.trans+lnWtgain+ Adult.diet
Fat.trans~Elytra+Ec.Weight.trans+lnWtgain +Adult.diet
lnWtgain~Elytra+Ec.Weight.trans+EndBBweight +Adult.diet
Ec.Weight.trans~EndBBweight+Developmenttime
Elytra~EndBBweight+Developmenttime
Developmenttime~EndBBweight

#covariances
lnProt~~Fat.trans
Elytra~~Ec.Weight.trans'
```

```
# Fit the model and check the summary

female.fat.full.model <- sem(Path.full.model.fat, data = femaledata,
    missing = "fiml")
summary(female.fat.full.model, standardized = TRUE)
```

```
## lavaan (0.5-18) converged normally after 119 iterations
##
##   Number of observations                            93
```

```
##
##   Number of missing patterns                         4
##
##   Estimator                                         ML
##   Minimum Function Test Statistic                8.459
##   Degrees of freedom                                 8
##   P-value (Chi-square)                           0.390
##
## Parameter estimates:
##
##   Information                                 Observed
##   Standard Errors                             Standard
##
##                    Estimate  Std.err  Z-value  P(>|z|)   Std.lv  Std.all
## Regressions:
##   lnProt ~
##     Elytra           -0.305    0.153   -1.998    0.046   -0.305   -0.342
##     Ec.Weght.trns     0.127    0.050    2.514    0.012    0.127    0.397
##     lnWtgain          0.223    0.151    1.470    0.142    0.223    0.203
##     Adult.diet        0.062    0.029    2.128    0.033    0.062    0.216
##   Fat.trans ~
##     Elytra            0.029    0.149    0.193    0.847    0.029    0.024
##     Ec.Weght.trns     0.100    0.049    2.032    0.042    0.100    0.234
##     lnWtgain          0.661    0.147    4.500    0.000    0.661    0.448
##     Adult.diet       -0.157    0.029   -5.483    0.000   -0.157   -0.405
##   lnWtgain ~
##     Elytra            0.396    0.101    3.922    0.000    0.396    0.487
##     Ec.Weght.trns     0.079    0.037    2.125    0.034    0.079    0.272
##     EndBBweight      -0.012    0.035   -0.339    0.735   -0.012   -0.031
##     Adult.diet        0.000    0.021    0.014    0.989    0.000    0.001
##   Ec.Weight.trans ~
##     EndBBweight       0.641    0.118    5.410    0.000    0.641    0.485
##     Developmenttm    -0.020    0.013   -1.476    0.140   -0.020   -0.132
##   Elytra ~
##     EndBBweight       0.184    0.044    4.222    0.000    0.184    0.389
##     Developmenttm    -0.012    0.005   -2.436    0.015   -0.012   -0.225
##   Developmenttime ~
##     EndBBweight      -1.432    0.917   -1.563    0.118   -1.432   -0.160
##
## Covariances:
##   lnProt ~~
##     Fat.trans        -0.001    0.002   -0.735    0.462   -0.001   -0.080
##   Ec.Weight.trans ~~
##     Elytra            0.036    0.007    5.308    0.000    0.036    0.659
##
## Intercepts:
##     lnProt            3.044    0.720    4.230    0.000    3.044   21.219
##     Fat.trans        -3.326    0.699   -4.762    0.000   -3.326  -17.242
##     lnWtgain          4.167    0.247   16.874    0.000    4.167   31.901
##     Ec.Weght.trns     2.940    0.448    6.557    0.000    2.940    6.537
##     Elytra            3.335    0.166   20.098    0.000    3.335   20.734
##     Developmenttm    31.446    1.314   23.936    0.000   31.446   10.323
##
## Variances:
```

```
##     lnProt          0.017    0.003                             0.017    0.831
##     Fat.trans       0.016    0.002                             0.016    0.433
##     lnWtgain        0.009    0.001                             0.009    0.513
##     Ec.Weght.trns   0.147    0.022                             0.147    0.727
##     Elytra          0.020    0.003                             0.020    0.769
##     Developmenttm   9.042    1.326                             9.042    0.974
```

7.2 Final model

This leads to a reduced model as follows:

```
#Specify the reduced model


Path.reduced.model.fat<-'#Regressions

Fat.trans~Ec.Weight.trans+lnWtgain +Adult.diet
lnWtgain~Elytra+Ec.Weight.trans
Ec.Weight.trans~EndBBweight
Elytra~EndBBweight

#covariances
Elytra~~Ec.Weight.trans'
```

```
# Fit the model and check the summary

female.fat.reduced.model <- sem(Path.reduced.model.fat, data = femaledata,
    missing = "fiml")
summary(female.fat.reduced.model, standardized = TRUE, fit.measures = TRUE)
```

```
## lavaan (0.5-18) converged normally after  73 iterations
##
##   Number of observations                            93
##
##   Number of missing patterns                         4
##
##   Estimator                                         ML
##   Minimum Function Test Statistic                6.024
##   Degrees of freedom                                 6
##   P-value (Chi-square)                           0.420
##
## Model test baseline model:
##
##   Minimum Function Test Statistic              236.467
##   Degrees of freedom                                14
##   P-value                                        0.000
##
## User model versus baseline model:
##
##   Comparative Fit Index (CFI)                    1.000
##   Tucker-Lewis Index (TLI)                       1.000
##
```

```
## Loglikelihood and Information Criteria:
##
##   Loglikelihood user model (H0)                 66.268
##   Loglikelihood unrestricted model (H1)         69.280
##
##   Number of free parameters                         16
##   Akaike (AIC)                                -100.536
##   Bayesian (BIC)                               -60.014
##   Sample-size adjusted Bayesian (BIC)         -110.523
##
## Root Mean Square Error of Approximation:
##
##   RMSEA                                          0.007
##   90 Percent Confidence Interval          0.000  0.135
##   P-value RMSEA <= 0.05                          0.563
##
## Standardized Root Mean Square Residual:
##
##   SRMR                                           0.037
##
## Parameter estimates:
##
##   Information                                 Observed
##   Standard Errors                             Standard
##
##                    Estimate  Std.err  Z-value  P(>|z|)   Std.lv  Std.all
## Regressions:
##   Fat.trans ~
##     Ec.Weght.trns     0.106    0.039    2.726    0.006    0.106    0.247
##     lnWtgain          0.673    0.135    4.967    0.000    0.673    0.456
##     Adult.diet       -0.156    0.028   -5.579    0.000   -0.156   -0.403
##   lnWtgain ~
##     Elytra            0.394    0.098    4.011    0.000    0.394    0.485
##     Ec.Weght.trns     0.075    0.034    2.170    0.030    0.075    0.258
##   Ec.Weight.trans ~
##     EndBBweight       0.669    0.118    5.654    0.000    0.669    0.506
##   Elytra ~
##     EndBBweight       0.202    0.044    4.544    0.000    0.202    0.426
##
## Covariances:
##   Ec.Weight.trans ~~
##     Elytra            0.038    0.007    5.357    0.000    0.038    0.669
##
## Intercepts:
##     Fat.trans        -3.316    0.696   -4.761    0.000   -3.316  -17.210
##     lnWtgain          4.171    0.243   17.155    0.000    4.171   31.944
##     Ec.Weght.trns     2.327    0.170   13.725    0.000    2.327    5.173
##     Elytra            2.960    0.064   46.509    0.000    2.960   18.406
##
## Variances:
##     Fat.trans         0.016    0.002                      0.016    0.434
##     lnWtgain          0.009    0.001                      0.009    0.515
##     Ec.Weght.trns     0.151    0.022                      0.151    0.744
##     Elytra            0.021    0.003                      0.021    0.818
```

Once again the fit measures are fine. The final path model looks like this.

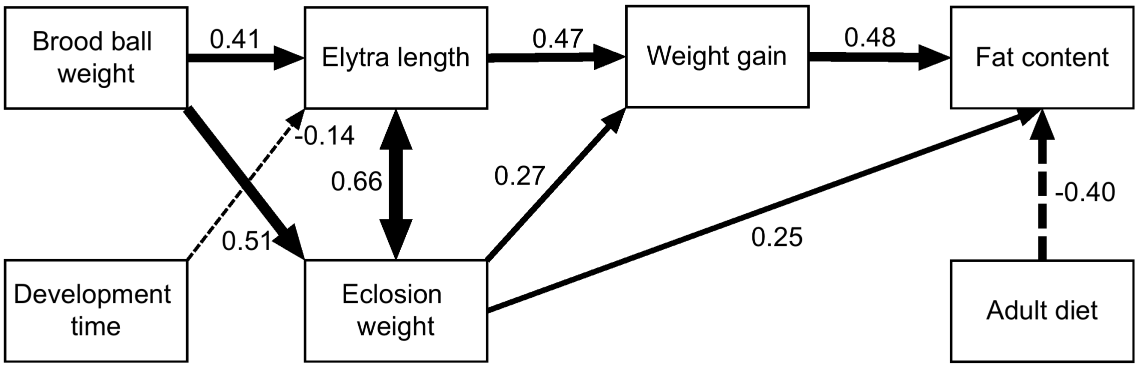

Figure J: Path diagram for the final model explaining fat content in females. Solid lines are positive relations, dashed lines negative, double headed arrows indicate correlation (i.e. no assumptions about causality), line width is proportional to the strength of the relationship.

7.3 Notes

Development time was very close to being retained in the final model as a predictor of elytra length (p=0.054, standardised regression coefficient = -0.127).

Haemolymph protein is not related to fat content in this case and was removed from the model because the fit was poor if it was retained, however note that haemolymph protein in females is significantly correlated with eclosion weight.

```
cor.test(femaledata$Ec.Weight.trans, femaledata$lnProt)
```

```
##
##  Pearson's product-moment correlation
##
## data:  femaledata$Ec.Weight.trans and femaledata$lnProt
## t = 2.536, df = 83, p-value = 0.01309
## alternative hypothesis: true correlation is not equal to 0
## 95 percent confidence interval:
##  0.05837398 0.45526668
## sample estimates:
##       cor
## 0.2681619
```

References

R Core Team. 2014. *R: A Language and Environment for Statistical Computing*. Vienna, Austria: R Foundation for Statistical Computing. http://www.R-project.org/.

Rosseel, Yves. 2012. "lavaan: An R Package for Structural Equation Modeling." *Journal of Statistical Software* 48 (2): 1–36. http://www.jstatsoft.org/v48/i02/.

Wickham, Hadley. 2009. *Ggplot2: Elegant Graphics for Data Analysis*. Springer New York. http://had.co.nz/ggplot2/book.
